# Nested Stochastic Block Models applied to the analysis of single cell data

**DOI:** 10.1186/s12859-021-04489-7

**Published:** 2021-11-30

**Authors:** Leonardo Morelli, Valentina Giansanti, Davide Cittaro

**Affiliations:** 1 Center for Omics Sciences, IRCCS San Raffaele Institute, Milan, Italy; 2 Università Vita-Salute San Raffaele, Milan, Italy; 3 Department of Informatics, Systems and Communication, University of Milano-Bicocca, Milan, Italy

## Abstract

Single cell profiling has been proven to be a powerful tool in molecular biology to understand the complex behaviours of heterogeneous system. The definition of the properties of single cells is the primary endpoint of such analysis, cells are typically clustered to underpin the common determinants that can be used to describe functional properties of the cell mixture under investigation. Several approaches have been proposed to identify cell clusters; while this is matter of active research, one popular approach is based on community detection in neighbourhood graphs by optimisation of modularity. In this paper we propose an alternative and principled solution to this problem, based on Stochastic Block Models. We show that such approach not only is suitable for identification of cell groups, it also provides a solid framework to perform other relevant tasks in single cell analysis, such as label transfer. To encourage the use of Stochastic Block Models, we developed a python library, schist, that is compatible with the popular scanpy framework.

**Supplementary Information:**

The online version contains supplementary material available at 10.1186/s12859-021-04489-7.

## Background

Transcriptome analysis at single cell level by RNA sequencing (scRNA-seq) is a technology growing in popularity and applications [1]. It has been applied to study the biology of complex tissues [2, 3], tumor dynamics [4–7], development [8, 9] and to describe whole organisms [10, 11].

A key step in the analysis of scRNA-seq data and, more in general, of single cell data, is the identification of cell populations, that is groups of cells sharing similar properties. Several approaches have been proposed to achieve this task, based on well established clustering techniques [12, 13], consensus clustering [14–16] and deep learning [17]; many more have been recently reviewed [18, 19] and benchmarked [20]. As the popularity of single cell analysis frameworks Seurat [21] and scanpy [22] raised, methods based instead on graph partitioning became the *de facto* standards. Such methods require the construction of a cell neighbourhood graph (e.g. by *k *Nearest Neighbours, *k*NN, or shared Nearest Neighbours, *s*NN). Encoding cell-to-cell similarities into graphs has practical advantages beyond clustering, as many algorithms for graph analysis can be applied and interpreted in a biological way. A notable example is the analysis of cell trajectories which can be derived from the analysis of Markov processes traversing the NN graph [23, 24]. In another context, computation of RNA moments in scRNA velocity is also based on the NN graph structure [25]. Arguably, the biggest utility of NN structure is the possibility to identify cell groups by partitioning the graph into communities; this is typically achieved using the Louvain method [26], a fast algorithm for optimisation of graph modularity. While fast, this method does not guarantee the identification of internally connected communities. To overcome its limits, a more recent approach, the Leiden algorithm [27], has been implemented and it has been quickly adopted in the analysis of single cell data, for example by scanpy [22] and PhenoGraph [28]. In addition to Newman’s modularity [29], other definitions currently used in single cell analysis make use of a resolution parameter [30, 31]. In lay terms, resolution works as a threshold on the density within communities: lowering the resolution results in less and sparser communities and *vice versa*. Identification of an appropriate resolution has been recognised as a major issue [32], also because it requires the definition of a mathematical property (clusters) over biological entities (the cell groups), with little formal description of the latter. In addition, the larger the dataset, the harder is to identify small cell groups, as a consequence of the well-known resolution limit [33]. Moreover, it has been demonstrated that random networks can have modularity [34] and its optimisation is incapable of separating actual structure from those arising simply of statistical fluctuations of the null model. Lastly, it is a common error to assume that the resolution parameter reflects a hierarchical structure of the communities in the graph when, in general, this is not rigorously true. Additional solutions to cell group identification from NN graphs have been proposed, introducing resampling techniques [35, 36] or clique analysis [37]. It has been proposed that high resolution clustering, e.g. obtained with Leiden or Louvain methods, can be refined in agglomerative way using machine learning techniques [38].

An alternative solution to community detection is the Stochastic Block Model, a generative model for graphs organised into communities [39]. In this scenario, identification of cell groups requires the estimation of the proper parameters underlying the observed NN graph. According to the microcanonical formulation [40], the parameters are partitions and the matrix of edge counts between them. Under this model, nodes belonging to the same group have the same probability to be connected together. It is possible to include node degree among the model parameters [41], to account for heterogeneity of degree distribution of real-world graphs. A Bayesian approach to infer parameters has been developed [42] and implemented in the graph-tool python library (https://graph-tool.skewed.de). There, a generative model of network ${\varvec{A}}$ has a probability $P(\varvec{A}|{\varvec{\theta }}, {\varvec{b}})$ where ${\varvec{\theta }}$ is the set of parameters and ${\varvec{b}}$ is the set of partitions. The likelihood of the network being generated by a given partition can be measured by the posterior probability

1 $$\begin{aligned} P({\varvec{b}} | {\varvec{A}}) = \frac{P({\varvec{A}}|{\varvec{\theta }}, {\varvec{b}})P({\varvec{\theta }}, {\varvec{b}})}{P({\varvec{A}})} \end{aligned}$$

and inference is performed by maximising the posterior probability. The numerator in Eq. 1 can be rewritten exponentiating the description length

2 $$\begin{aligned} \Sigma = -\ln P({\varvec{A}}|{\varvec{\theta }}, {\varvec{b}}) - \ln P({\varvec{\theta }}, {\varvec{b}}) \end{aligned}$$

so that inference is performed by minimising the information required to describe the data (Occam’s razor); graph-tool is able to efficiently do this by a Markov Chain Monte Carlo approach [43]. SBM itself may fail to identify small groups in large graphs, hence hierarchical formulation has been proposed [44]. Under this model, communities are agglomerated at a higher level in a block multigraph, also modelled using SBM. This process is repeated recursively until a graph with a single block is reached, creating a nested Stochastic Block Model (nSBM).

In this work we propose nSBM for the analysis of single cell data, in particular scRNA-seq data. This approach identifies cell groups in a statistical robust way and, moreover, it is able to determine the likelihood of the grouping, thus allowing model selection. In addition, it is possible to measure the confidence of assignment to groups, a measure that can be exploited in various analysis tasks.

We developed schist (https://github.com/dawe/schist), a python library compatible with scanpy, to facilitate the adoption of Stochastic Block Models in single-cell analysis.

## Results

### Overview of schist

schist is a convenient wrapper to the graph-tool python library, written in python and designed to be used with scanpy. The most prominent function is schist.inference.nested_model() which takes a AnnData object as input and fits a nested Stochastic Block Model on the *k*NN graph built with scanpy functions (e.g. scanpy.tools.neighbors()). When launched with default parameters, schist fits a model which maximises the posterior probability of having a set of cell groups (or blocks) given a graph. schist then annotates cells in the data object with all the groups found at each level of a hierarchy. Given the large size of the NN graph in real-world experiments, it is possible that a single solution represents local minima of the fitting process. In addition, it is possible that multiple solutions are equally acceptable to represent the graph partitioning and a better description is given by the consensus over such solutions [45]. To overcome these issues, schist fits multiple instances in parallel and returns the inferred consensus model, alongside the marginal probabilities for each cell to belong to a specific group (*cell marginals*). Moreover, the Stochastic Block Model has no constraints on what type of modular structure is fitted, meaning that groups are not necessarily identified only by assortativity (i.e. cells are mostly connected within the same group). When assortativity is thought to be the dominant pattern another model (the Planted Partition Block Model, PPBM [46]), also implemented in schist, is better suited to find statistically significant assortative communities, also eliminating the need to set a resolution parameter as required in standard community detection by maximisation of modularity.

### Analysis of the impact of noise

**Fig. 1:**
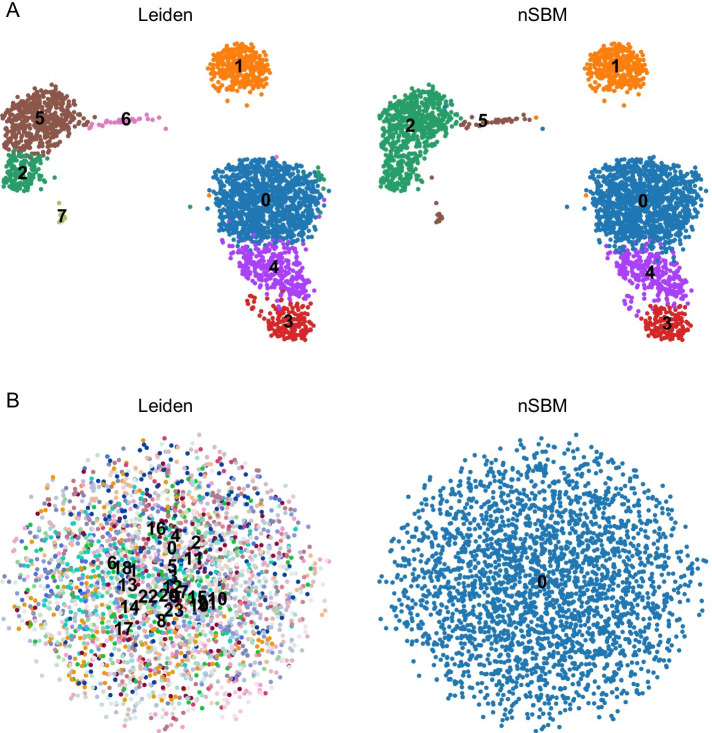
Modeling randomised data. **A** UMAP embedding of 3k PBMC after standard processing with Leiden approach (left) or nSBM (right). Cell grouping is consistent for both the approaches (Adjusted Rand Index $ARI=0.869$). **B** UMAP embedding of the same data after randomisation of *k*NN graph edges. In this case schist does not return any cell grouping, while optimisation of modularity finds up to 24 different cell groups

One of the most relevant difference between the SBM and other methods to cluster single cells is that it relies on robust statistical modelling. In this sense, the number of groups identified strictly mirrors the amount of information contained in the data. An important consequence is that absence of information (i.e. maximal entropy) can be properly handled. To show this property we performed a simple experiment on a randomised *k*NN graph. We collected data for 3k PBMC (available as preprocessed data in scanpy, Fig. 1A) and shuffled the edges of the prebuilt *k*NN graph, this to keep the general graph properties unchanged. We tested that the degree distribution does not change after randomisation (Kolmogorov-Smirnov $D=0.0733$, $p=0.703$). We found that the default strategy, based on maximisation of modularity, identifies 24 cell groups at default resolution, whereas schist does not identify any cell group, at level 0 (Fig. 1B).

Only by reducing resolution to $\gamma < 0.6$ we were able to obtain a single partition by modularity (Additional file 1: Fig. S1). Of course, this experiment is a deliberate extreme case. The quality of grouping proposed by a standard approach can be disputed in many ways, and the UMAP embedding indeed reflects the absence of any information. Nevertheless, real-world data may include an unknown amount of random noise. Hence, it is important to identify cell groups that are not artefacts arising from processing and that do reflect the information contained in the dataset. To understand the impact of random noise on structured data, we considered the same PBMC dataset and added white noise to the normalised counts at increasing levels of $\sigma$, ensuring that the noise level is modelled after the feature-wise distribution of detected genes. We then compared partitions to the original annotation by Adjusted Rand Index (*ARI*), we found that schist is more robust to perturbations and that, again, optimising the modularity results in overestimation of the number of communities at high noise levels (Additional file 1: Table S1). Of note, the concordance with original annotations drops at $\sigma \ge 1.5$ for both the approaches.

### schist correctly identifies cell populations

**Fig. 2:**
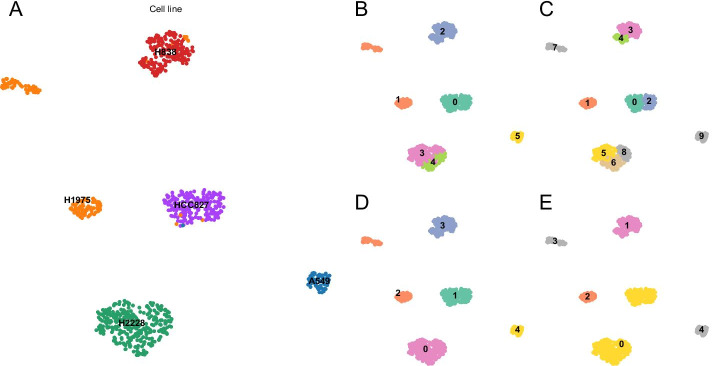
Analysis of a mixture of 5 known cell lines. **A** UMAP embedding coloured and labeled by cell line identity. H1975 cells (orange) show two distinct groups reflecting an internal heterogeneity. **B** UMAP embedding showing cells coloured by level 1 of the hierarchy proposed by the nested Stochastic Block Model. **C** UMAP embedding showing cells coloured according to the Leiden method at resolution $\gamma = 1$. **D** UMAP embedding coloured by the classification made by SCCAF when partitions in (**B**) are used. **E** UMAP embedding coloured by the classification made by SCCAF when partitions in (**C**) are used

To benchmark schist, we tested it on scRNA-seq mixology data [47], a dataset explicitly developed to benchmark single cell analysis tools without the need to simulate data. In particular, we used the mixture of 5 cell lines profiled with Chromium 10x platform. At a first evaluation of the UMAP embedding, all lines appear well separated. Only the lung cancer line H1975 shows a considerable degree of heterogeneity and appears to be split into two cell groups (Fig. 2A). Using default parameters, schist is able to identify correct cell groups ($ARI=0.829$), with a further split in H2228 cell line (Fig. 2B), whereas Leiden method clusters the dataset into 10 groups ($ARI=0.549$, Fig. 2C). schist correctly identifies H1975 groups as a single entity at level 1 of the nSBM hierarchy. We then sought to check if an independent agglomerative method, SCCAF [38], was able to recover cell line groupings starting from both partition schemes. Given the ground truth, the cell lines, SCCAF is able to assess the maximal accuracy that can be achieved in the dataset (0.992). When trained with this target accuracy, SCCAF precisely reconstructs the original cell line annotations starting from schist partitions with high accuracy (Fig. 2D). When Leiden partitions are set as input, SCCAF merges H2228 and HCC827 cells into a single cluster and keeps H1975 cells split into two groups (Fig. 2E), highlighting potential limitations of this approach.

In another experiment, we analysed data from the Tabula Muris project [48] mixing four different tissues as previously performed [49] (i.e. skin, spleen, large intestine and brain, Additional file 1: Fig. S2A). In this experiment we expect higher heterogeneity than controlled cell lines, however schist is able to correctly identify the original tissues (Additional file 1: Fig. S2C), which are again almost perfectly classified after SCCAF is applied (Additional file 1: Fig. S2D). Similarly to the cell line experiment, optimisation of modularity isolates cell clumps evident in UMAP embedding (Additional file 1: Fig. S2E) which could not be correctly merged after SCCAF iteration (Additional file 1: Fig.S2F). In all, these data support the suitability of schist, hence of nested Stochastic Block Models, for cell group identification in single cell studies.

### Hierarchy modelling complies with biological properties

When grouping is performed by optimisation of modularity, there is often the implicit assumption that the resolution parameter reflects a hierarchical structure of the graph, i.e. communities are consistently grouped at lower resolutions. Not only this assumption is wrong, but it may also lead to spurious groupings in real experiments, whereas a nSBM inherently encodes hierarchies by merging communities in a tree. The improper use of resolution parameter may lead to two types of errors: grouping of cells that are in fact distinct and creating an inconsistent hierarchy.

**Fig. 3:**
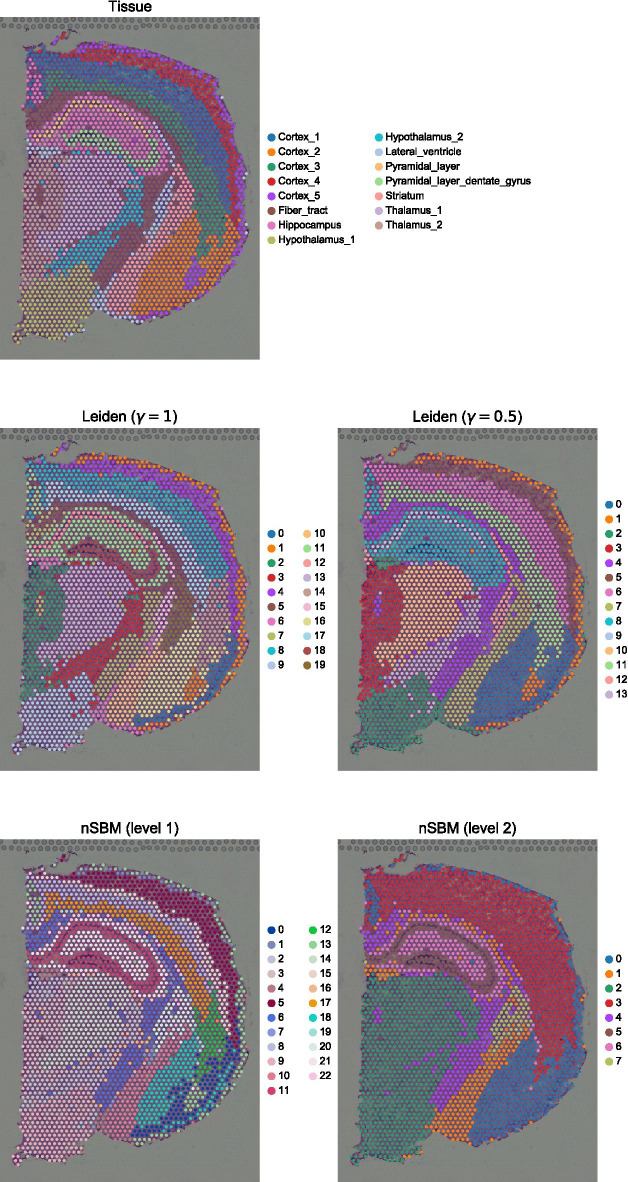
Analysis of spatial transcriptomics of a coronal section of mouse brain by Visium H&E. In the first panel, original tissue annotation is given. Tissues are well defined at default resolution for the standard approach. When resolution is decreased to $\gamma = 0.5$, cells are aggregated breaking the histological types, e.g. cells from the dentate gyrus are merged to the hippocampus. When a nSBM is applied, the structure of the pyramidal layer is maintained at different levels of the hierarchy

To show this we took advantage of public spatial RNA dataset of a coronal section of murine brain tissue profiled with 10X Visium H&E technology [50], as provided by the recently introduced package SquidPy [51]. We chose to stick to the given tissue annotation by the package authors. At default resolution, Leiden clustering resolves the tissue structure, as does the first level of the nSBM hierarchy (Fig. 3). When resolution is decreased (e.g. $\gamma = 0.5$), the dentate gyrus is incorrectly merged to the hippocampus, whereas schist correctly identifies the pyramidal layer.

**Fig. 4:**
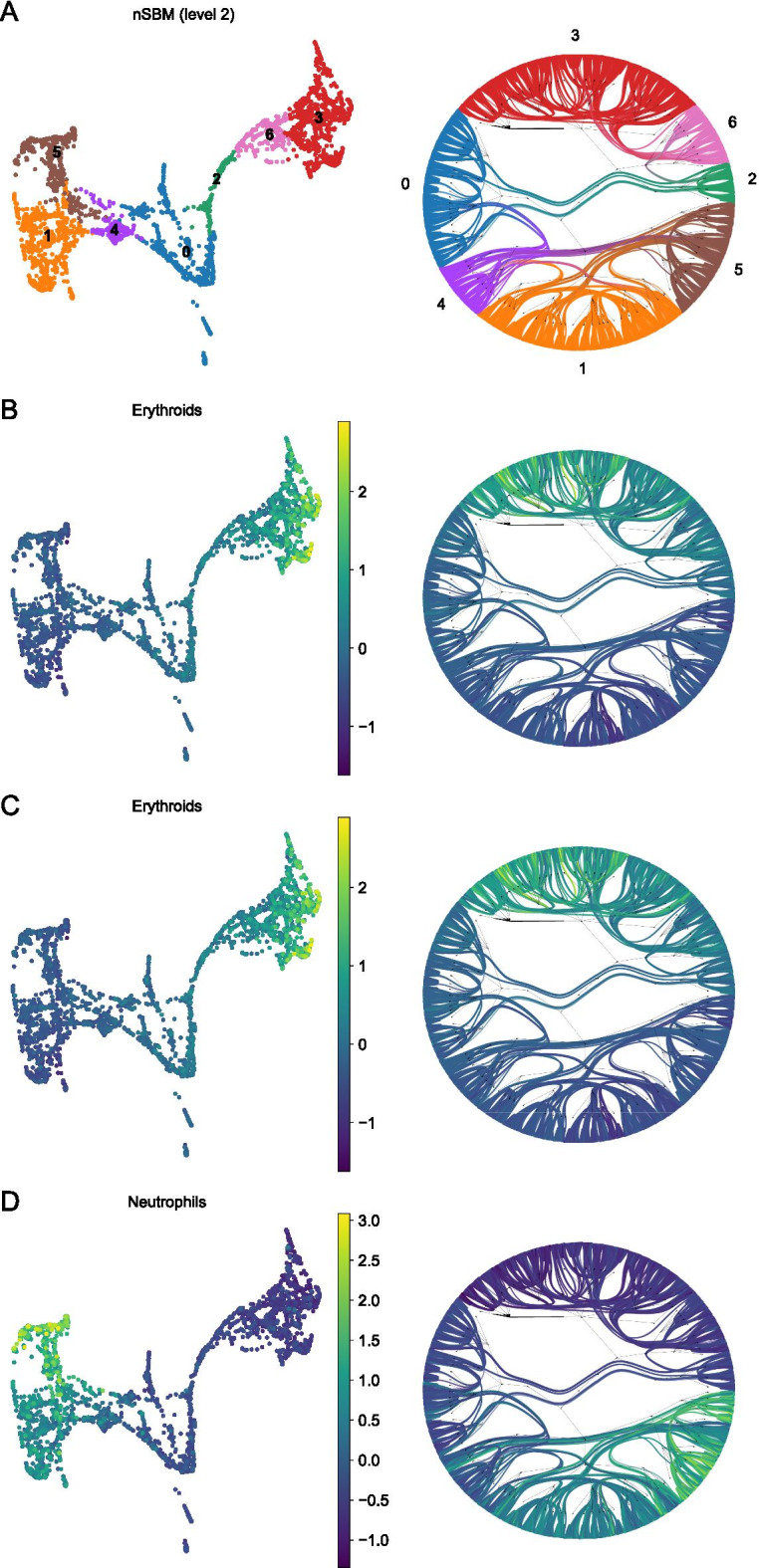
Analysis of hematopoietic differentiation. Each panel presents a low dimensional embedding of single cells next to a radial tree representation of the nSBM hierarchy. Cells are coloured according to groupings at level 2 of the hierarchy, group 0 marks the most primitive population (**A**). In subsequent panels, cells are coloured using a signature of Erythroid lineage (**B**), Monocytes (**C**) or Neutrophils (**D**)

In another context, we tested the effect on the interpretation of the hierarchy varying the resolution parameter. We analysed data for hematopoietic differentiation [52], previously used to benchmark the consistency of cell grouping with differentiation trajectories by graph abstraction [53] (Additional file 1: Fig. S3A). Data show three major branchings (Erythroids, Neutrophils and Monocytes) stemming from the progenitor cells, mostly recapitulated by level 2 of the hierarchy computed by schist (Fig. 4). Not only the hierarchic model recapitulates the branching trajectories, also the cell groups appear to be consistent with the estimated pseudotime (Additional file 1: Fig. S3B). Conversely, the Leiden method at default resolution identified 24 groups. By lowering the $\gamma$ parameter we observed cell groups that merge and split at different resolutions disrupting the hierarchy (Additional file 1: Fig. S4).

In all, these data show that the common intuition that $\gamma$ parameter acts as a thresholding factor over a hierarchy is wrong. Not only the hierarchy is not conserved, but also very different cell types may be mixed in spurious clusters. By using nSBM, schist is able to represent hierarchical relations in appropriate way. Moreover, the hierarchy appears to be more robust in aggregating different cell types at coarser scales.

### Cell marginals can be used to assess the data quality

By computing the consensus among multiple models, schist returns the marginal probability for each cell to belong to a specific cluster at each level of the hierarchy. Ideally, all cells should always be assigned with $p=1$ to a cluster. When the uncertainty is maximal, cells are assigned to clusters randomly with $p=1/B_i$, where $B_i$ is the number of groups for the *i*-th level in the hierarchy. We sought to check if these probabilities could be interpreted in terms of data quality.

We devised a simple metric, *cell stability*, that is defined by the fraction of levels for which the marginal probability is higher than $1-1/B_i$. To do so, we only consider levels with at least two groups, hence excluding the root of the tree. We tested this metric on four datasets from [54] with different quality levels (iCELL8, MARS-seq, 10XV3 and Quartsz-seq2) (Additional file 1: Fig. S5). By taking a summary metric, e.g. the mean ${\overline{S}}$ or the fraction of cells with $S>0.5$, we observed that it correlates with the data quality (Table 1).

**Table 1:** Cell stability as indicator of data quality

Dataset	${\overline{S}}$	$S > .5$
iCELL8 [54]	0.368	0.312
MARS-seq [54]	0.579	0.536
Chromium 10x [54]	0.716	0.728
Quartz-seq2 [54]	0.705	0.739

Table shows summary metrics derived from the Cell Stability calculated for various datasets. ${\overline{S}}$ is the average Cell Stability over all cells, $S>.5$ indicates the fraction of cells with Cell Stability higher than 0.5

These data suggest that measures of uncertainty of cell clustering can be useful for general quality control assessment. In addition to this, we foresee they could be used to isolate cells with specific patterns.

### Cell affinities can be used for label transfer

The modelling approach we adopted allows the estimation of the information required to describe a graph given any partitioning scheme, not limited to the solution given by the model itself. Differences in entropy can be used to perform model selection, hence we can choose which model better describes the data. We sought to exploit this property to address the task of annotating cells according to a reference sample. To this end we analysed datasets from [54], which includes mixtures of human PBMC and HEK293T cells profiled with various technologies. We chose cells profiled with 10X V3 platform as reference dataset and performed annotation on cells profiled with Quartz-seq2 or MARS-seq. These are at the extremes of the capability to distinguish cell types, so they provide good benchmark configurations for this task.

**Fig. 5:**
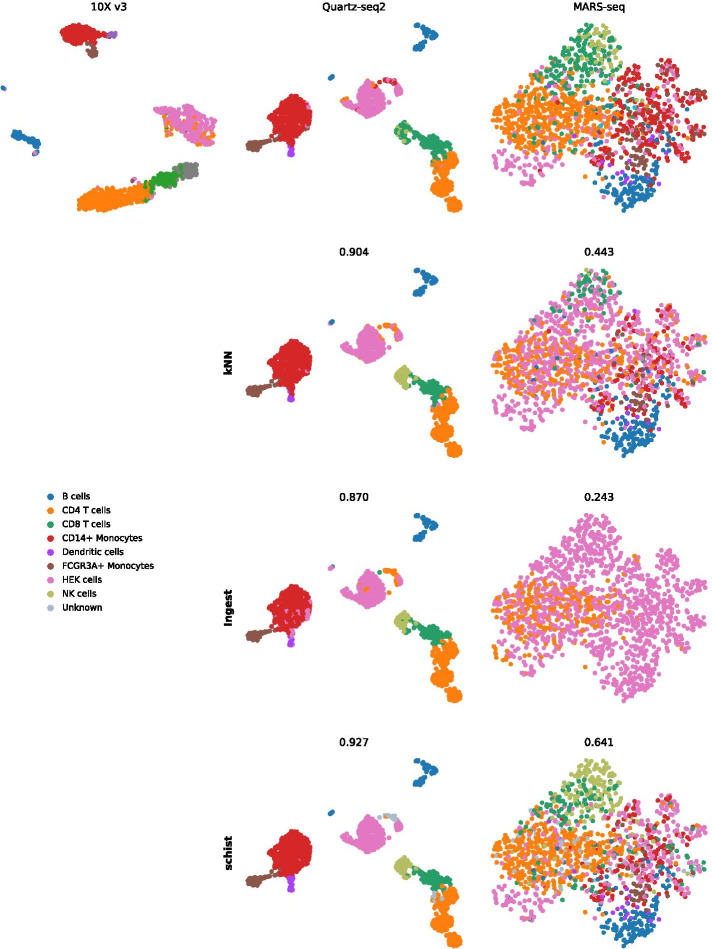
Label transfer using SBM. The first line reports UMAP embeddings for datasets profiled with Chromium 10X V3, Quartz-seq2 and MARS-seq, each annotated by known cell types. Quartz-seq2 and MARS-seq were reannotated using *k*NN method, scanpy.tools.ingest() or schist. The accuracy of each label transfer task is reported above the corresponding UMAP

After preprocessing raw data according to the parameters given in [54], we integrated each dataset with 10XV3 into a unified representation using Harmony [55], and computed the *k*NN graph. In each merged dataset, we retained cell type annotations for 10X cells, while we assigned a “Unknown” label to all cells derived from the other technology (i.e. MARS-seq or Quartz-seq2). We then calculated the *cell affinity* matrix, that is we computed the difference in entropy that can be observed by assigning each cell to each annotation cluster, this being either one of the original cell types or “Unknown”. Once the matrix has been computed, each cell from the query data is assigned to the group with the highest likelihood. The rationale behind this approach is that if cells belong to the same annotation group, then more information is required to describe the graph if they were annotated as different cell types; hence, cells from the query datasets should retain their “Unknown” label if and only if there is not enough evidence to associate them to another group. We compared the accuracy of the outcome to *k*NN classification, given by the closest entry in the *k*NN graph, and to ingest, a tool included in scanpy based on *k*NN classification of UMAP embeddings. Analysis of a well defined dataset, such as Quartz-seq2, reveals that the three approaches are equally good in classifying unknown cells (Fig. 5, central column), with accuracies ranging from .870 to .927. When data are noisy, instead, *k*NN-based methods show low accuracy and a tendency to assign the most represented cell group (HEK293T) to the unlabelled cells. This misannotation is particular evident for ingest, in which only CD4 T cells and HEK cells are transferred, resulting in the lowest accuracy (0.243). Conversely, schist is able to assign correct labels with higher accuracy (0.641). Moreover, *k*NN methods assign a label to each cell, whereas schist does not relabel cells if there are no sufficient evidence (e.g. the “Unknown” state is the most likely). Interestingly, we found that for the largest part of cells without assigned label, the second choice by affinity ranking was indeed the appropriate one (Additional file 1: Fig. S6).

### Choice of an optimal hierarchy level

schist fits a hierarchical model of communities into a graph. When it comes to analysis of single cell data, it means that the cells are best described by the hierarchy itself and that cells *can* be grouped consistently at each level of the tree. In addition, the size of groups at the deepest level scales as $O(N/\log {N})$ [44], where *N* is the number of cells. Given the current throughput in single cell experiments ($\sim$10k cells), the number of groups is difficult to handle. For this reason, in most of single cell experiments, it is preferable to identify an optimal level of the hierarchy that best resembles the cell properties at the scale they can be validated.

A possible strategy is based on Random Matrix Theory, as suggested by the authors of the SC3 package [15], for which a suitable number of clusters, ${\hat{k}}$, is determined by the number of eigenvalues of the ${\varvec{Z}}^\top {\varvec{Z}}$ matrix (where ${\varvec{Z}}$ is the normalised count matrix) significantly different at $p<.001$ from the appropriate Tracy-Widom distribution. According to this strategy, the optimal level $i^k$ is the one that minimises the number of partitions and ${\hat{k}}$:

3 $$\begin{aligned} i^k = \underset{x}{\mathrm {argmin}} |B_x - {\hat{k}}| \end{aligned}$$

where $B_x$ is the number of non empty partitions at level *x*.

An alternative strategy is to evaluate the behaviour of modularity at different hierarchy levels. While schist does not optimise the graph modularity *Q*, we observed that this tends to be maximal for the level better describing known cell populations, so the optimal level $i^Q$ is

4 $$\begin{aligned} i^Q = \underset{x}{\mathrm {argmax}} |Q_x| \end{aligned}$$

Where $Q_x$ is modularity at level *x*. We collected values arising from both the approaches for some datasets used in this work (Table 2 and Additional file 1: Fig. S7)

**Table 2:** Selection of the optimal level in the nSBM hierarchy

Dataset	Cells	*D*	${\hat{k}}$	$i^k$	$B_k$	$i^Q$	$B_Q$
sc-mixology [47]	860	5	21	1	6	1	6
Chromium 10x [54]	1523	8	43	0	58	1	13
Quartz-seq2 [54]	1266	8	37	0	62	1	12
MARS-seq [54]	1401	9	9	1	16	1	16
iCELL8 [54]	1830	9	20	1	21	2	6
Mouse brain [50]	2688	15	8	2	8	1	23
Planaria [10]	21,612	$51^*$	34	2	22	3	10

For each dataset we report the number of groups *D* that were given by the authors. The optimal level selection should recover a number of groups in the order of magnitude of *D*. Value of *D* in Planaria dataset is derived from manual curation of Louvain clustering. ${\hat{k}}$: number of groups according to RMT, $i^k$: level selected according to RMT criterion, $B_k$: number of partitions at level $i^k$, $i^Q$: level at which modularity is maximal, $B_Q$: number of groups at level $i^Q$

**Fig. 6:**
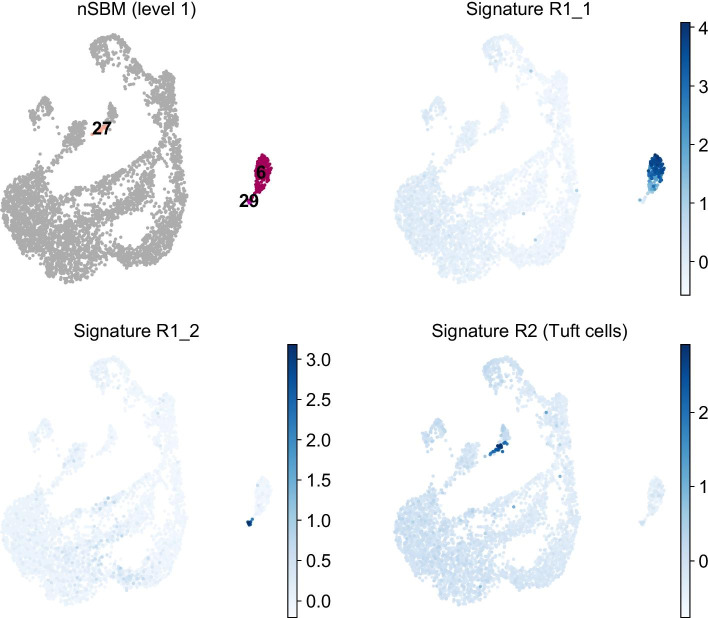
Identification of rare populations. UMAP embeddings of mouse crypt cells highlighting the groups found at level 1 of nSBM hierarchy corresponding to rare populations identified by GapClust [57]. Each rare population is colored by a signature calculated from published gene lists (see methods)

As expected, the larger the network, the higher the optimal level. For relatively small datasets (i.e. less than 10k cells), the first level of the hierarchy contains a number of groups in line with how many observable populations are. Notwithstanding, cell groups identified at leach level may have a biological interpretation. In particular, groups at deepest levels (0 or 1) may be relevant when studying rare populations. For example, in the hematopoiesis dataset shown in Additional file 1: Fig. S3A, groups 11 (DC) and 19 (Lymph) cannot be distinguished from nSBM level 1 and up; a closer investigation to level 0, however, revealed that these cells are clearly separated (Additional file 1: Fig. S3D). To better understand the role of deepest levels, we performed an additional analysis of a single cell dataset of mouse crypt cells [56], which was also covered in a recent paper proposing GapClust as an optimal approach to identify rare cell populations [57]. We sought to identify the four rare populations identified by GapClust. We could distinguish all but the erythrocyte group (R3) at level 1 of the hierarchy (Fig. 6 and S8), suggesting that exploring nSBM levels with appropriate community size is a valid method to spot rare populations. Of note, modularity optimisation could not pinpoint Tuft cells in appropriate way (Additional file 1: Fig. S8C), not even at high resolution, hence prompting the development of specific approaches such as GapClust.

As the size and number of communities is strictly dependent on the *k*NN graph generation, we investigated how different parameters (i.e. number of principal components and number of neighbors) affect the partition structure (Additional file 1: Fig. S9). We found, as a general pattern, that increasing the number of neighbors results in more granular structure at level 0, with different solutions being consistent (Additional file 1: Fig. S10), suggesting that higher number of neighbors provides richer description of the dataset. The number of PCs used to evaluate cell-to-cell distance influences the variability of community sizes; the consistency among different solutions is high when a sufficient number of PCs is chosen, data suggest that for large datasets more PCs should be included to include adequate fraction of overall variability.

### Analysis of runtimes

Minimisation of a nSBM is a process that requires a large amount of computational resources. While the underlying graph-tool library is efficient in exploring the solution space using a multiflip MCMC sampling strategy, the number of required iterations before convergence is considerable and the running time scales linearly with the number of edges. Moreover, to collect a consensus partition, we minimise multiple models (default: 100) that need to be averaged. To give a reference, we report runtimes for some example datasets in Table 3 on a commodity hardware (Intel i7@2.8 GHz, 32 GB RAM). Compared to Leiden approach, nSBM requires at best $\sim 6\times$ times more, and $\sim 30\times$ at worst. A reasonably fast alternative to the nSBM is the Planted Partition Block Model (PPBM), for which we also report runtimes. The PPBM [46] is able to find statistically significant assortative modules and eliminates the resolution parameter; differently from nSBM, PPBM is not hierarchic.

**Table 3:** Time required to run different partitioning strategies implemented in schist on various datasets

Dataset	Cells	Edges	Leiden	PPBM	nSBM
sc-mixology [47]	860	9186	00:06	00:13	00:36
Quartz-seq2 [54]	1266	14,603	00:10	00:19	00:45
MARS-seq [54]	1401	21,756	00:20	00:34	02:14
iCELL8 [54]	1830	30,636	00:23	00:40	03:02
Chromium 10x [54]	1523	21,447	00:14	00:26	01:07
Hematopoiesis [52]	2730	15,444	00:37	01:27	05:52
Mouse Cortex [58]	3005	54,460	00:59	00:53	07:32
Endocrinogenesis [59]	3696	74,670	01:15	01:29	10:56
Baron Pancreas [60]	8569	294,480	03:51	07:35	1:33:40
Airzani Liver [61]	10,368	354,440	04:13	09:47	1:42:23
Tabula Muris [48]	12,434	265,610	03:07	10:00	1:23:35
Planaria [10]	21,612	173,667	05:41	13:52	1:20:40

All approaches fit 100 models. Number of nodes and edges refer to the structure of the *k*NN graph as built by scanpy. Times are expressed in MM:SS

## Conclusions

Identification of cells sharing similar properties in single cell experiments is of paramount importance. A large number of approaches have been described, although the standardisation of analysis pipelines converged to methods that are based on modularity optimisation. We tackled the biological problem using a different approach, nSBM, which has several advantages over existing techniques. As random data may have modular structure [34], an important property of our approach is that it does not overfit data by finding partitions when, in fact, there are not. Another important advantage is that the hierarchical definition of cell groups eliminates the choice of an arbitrary threshold on clustering resolution. In addition, we showed that the hierarchy itself could have a biological interpretation, implying that the hierarchical model is a valid representation of the cell ensemble. We performed experiments to evaluated the impact of parameters to build the *k*NN graph on the final partitions. We found that our solutions were consistent across parameters; we also found that the more information is included during graph generation, the more granular the final description. Our results suggest that the number of principal components used to evaluate the cell-to-cell distance may have an impact on the final results and that the number of components to include depends on the data size and heterogeneity; while intuitive, this finding is in contrast with what has been observed for other PCA-based methods [18], whereas has an impact on probabilistic methods [49].

The Bayesian formulation of Stochastic Block Models provides the possibility to perform inference on a graph for any partition configuration, thus allowing reliable model selection using an interpretable measure, entropy. We exploited this property to perform label transfer with high accuracy and with the possibility to discard cells with unreliable assignments. In all, schist facilitates the adoption of nSBM by the bioinformatics community and exposes a robust framework to perform tasks that go beyond the principled identification of cell clusters.

The major drawback of adopting this strategy is the substantial increase of runtimes. As observed, model minimisation is many times slower than the extremely fast Leiden approach. It should be noted that schist initialises multiple models that are treated by multiple concurrent processes. graph-tool itself supports CPU-level parallelisation for some of its tasks. These optimisations are well suited for clustered computing infrastructure. Further development, possibly including GPU-level parallelisation, is surely required to accomodate the large size of datasets that are being produced.

## Materials and methods

Unless differently stated, all the analysis were produced using scanpy v1.7.1 [22] and schist v0.7.6 and the corresponding dependencies. All models were initialised 100 times, herein including Leiden partitioning for which we also calculated the consensus partition.

### Analysis of randomized data

Data were retrieved in scanpy environment using scanpy.datasets.pbmc3k_processed() function. The random *k*NN graph was obtained shuffling the node labels of each edge. UMAP embedding was recomputed after randomisation using the shuffled graph. To generate data with white noise we computed the genewise means ($\mu _g$) and standard deviations ($\sigma _g$) of log-normalized counts excluding 0 values. We generated random values using $\mu _g$ and $k\sigma _g$, $k \in \{0.5, 1, 1.5, 2\}$, and added to original expression values.

### Analysis of cell mixtures

Data and metadata for five cell mixture profiled by Chromium 10x were downloaded from the sc-mixology repository (https://github.com/LuyiTian/sc_mixology). Cells with less than 200 genes were excluded, as genes detected in less than 3 cells. Cells with less than 5% of mitochondrial genes were retained for subsequent analysis. Data were normalised and log-transformed; number of genes and percentage of mitochondrial genes were regressed out. *k*NN graph was built with default parameters (50 components and 15 nearest neighbours). Data were assessed by SCCAF using cell line annotation. Mean cross-validated accuracy was set as target for all the models.

### Analysis of Tabula Muris data

Data for FACS isolated cells sequenced with Smart-seq2 were downloaded from the Tabula Muris consortium [49] (https://doi.org/10.6084/m9.figshare.5975392), analysis was restricted to Skin, Spleen, Large Intestine and Brain-Myeloid count matrices. Cells with less than 200 genes were excluded, as genes detected in less than 3 cells. Data were normalised and log-transformed. Merged data were then processed using Harmony [55] by the scanpy.external.pp.harmony_integrate() function with default parameters. *k*NN graph was built on integrated data using 50 components and 30 nearest neighbours. Data were assessed by SCCAF using tissue annotation. Mean cross-validated accuracy was set as target for all the models.

### Analysis of visium H&E data

Data were retrieved using squidpy.datasets.visium_hne_adata() built-in function, without further processing. Leiden clustering was performed using schist.inference.leiden() function, allowing for 100 initialisations, with resolutions $\gamma = 1$ and $\gamma = 0.5$.

### Analysis of hematopoietic differentiation

Data were retrieved using scanpy’s built-in functions and were processed as in [53], except for *k*NN graph built using 30 principal components, 30 neighbours and diffmap as embedding. Gene signatures were calculated with scanpy.tools.score_genes() using the following gene lists

- Erythroids: Gata1, Klf1, Epor, Gypa, Hba-a2, Hba-a1, Spi1
- Neutrophils, Elane, Cebpe, Ctsg, Mpo, Gfi1
- Monocytes, Irf8, Csf1r, Ctsg, Mpo

### Processing of PBMC data from various platforms

Count matrices were downloaded from GEO using the following accession numbers: GSE133535 (Chromium 10Xv3), GSE133543 (Quartz-seq2), GSE133542 (MARS-seq) and GSE133541 (iCELL8). Data were processed according to the methods in the original paper [54]. Briefly, cells with less than 10,000 total number of reads as well as the cells having less than 65% of the reads mapped to their reference genome were discarded. Cells in the 95th percentile of the number of genes/cell and those having less than 25% mitochondrial gene content were included in the downstream analyses. Genes that were expressed in less than five cells were removed. Data were normalised and log-transformed, highly variable genes were detected at minimal dispersion equal to 0.5. Neighbourhood graph was built using 30 principal components and 20 neighbours.

### Analysis of crypt cells data

Count matrix for untreated crypt cells (GSM3308718) was downloaded from GEO. Cells with less than 200 genes and genes detected in less than 2 cells were excluded from the analysis. After normalization and log-transformation, highly variable genes were selected with a cutoff on the mean expression equal to 0.05. Rare subpopulations were first highlighted with scanpy.tools.score_genes() using signatures published in [57]:

- R1_1: Cd8a, Cd3g, Ccl5, Gzma, Gzmb, RGs1, Nkg7, Cd7, Fcer1g
- R1_2: H2-Aa, H2-Ab1, H2-Eb1, Cd74, Ly6d, Ebf1, Cd79a, Mef2c
- R2: Krt18, Cd24a, Adh1, Cystm1, Aldh2, Dclk1, Sh2d6, Rgs13, Hck, Trpm5
- R3: Alas2, Hbb-bs, Hba-a1, Hbb-bt

### Label transfer

Processed data for MARS-seq or Quart-seq2 platforms were merged to data for 10X V3. Merged data were then processed using Harmony [55] by the scanpy.external.pp.harmony_integrate() function with default parameters. Cells not belonging to the 10X data were assigned an “Unknown” label. We calculated cell affinity to each annotation label using schist.tl.calculate_affinity() function. We assigned the most affine annotation only to “Unknown” cells. For *k*NN-based procedure, we built a *k*NN graph on the merged data using pynndescent library on the 10XV3 subset of cells in the merged data, then we assigned “Unknown” cells to the closest entry in the graph. Assignment by scanpy.tools.ingest() was performed using default parameters.

## Supplementary Information

**Additional file 1**. Supplementary Table S1; Supplementary Figures S1–S10.

## Data Availability

No datasets were generated in the current study. The original third-party datasets that were analysed are included in the corresponding publications [10, 47, 52, 54, 58–61]
