# Supplementary material for: Nested Stochastic Block Models applied to the analysis of single cell data
Source: BMC Bioinformatics. 2021 Nov 30;22:576. doi: 10.1186/s12859-021-04489-7 (PMC8630903; doi:10.1186/s12859-021-04489-7)
Supplement: Supplementary file 1 — Additional file 1. Supplementary Table S1; Supplementary Figures S1–S10. [file 12859_2021_4489_MOESM1_ESM.pdf]

Nested Stochastic Block Models Applied to the Analysis of Single Cell Data

Leonardo Morelli[1,2], Valentina Giansanti[1,3], and Davide Cittaro[1]

[1]Center for Omics Sciences, IRCCS San Raffaele Institute, Milan, Italy
[2]Università Vita-Salute San Raffaele, Milan, Italy
[3]Department of Informatics, Systems and Communication, University of Milano-Bicocca, Milan, Italy

November 29, 2021

Supplementary tables

Noise level (k)	Leiden		nSBM level 1	
	ARI	*N*	*ARI*	*N*
–	0.9228	8	0.8944	6
0.5	0.6249	10	0.7509	10
1.0	0.5966	7	0.7269	6
1.5	0.2833	9	0.3029	3
2.0	0.1405	12	0.1961	2

TABLE S1: Effect of random noise. The table shows performance of Leiden approach and nested Stochastic Block Model in recognising annotated population in PBMC 3k data when increasing level of noise are added to log-normalized counts. The k parameter multiplies the σ, estimated from data, to generate random counts. *ARI*: Adjusted Rand Index, *N*: number of communities.

Supplementary figures

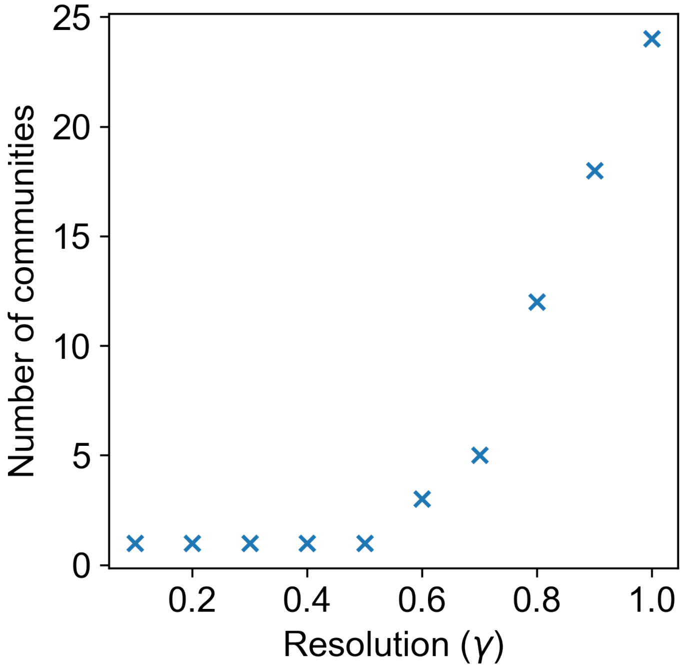

Figure S1: Decreasing the resolution parameter γ results in lower number of communities also in the random graph generated from PBMC 3k data. At $\gamma < 0.6$, Leiden approach correctly identifies one single community.

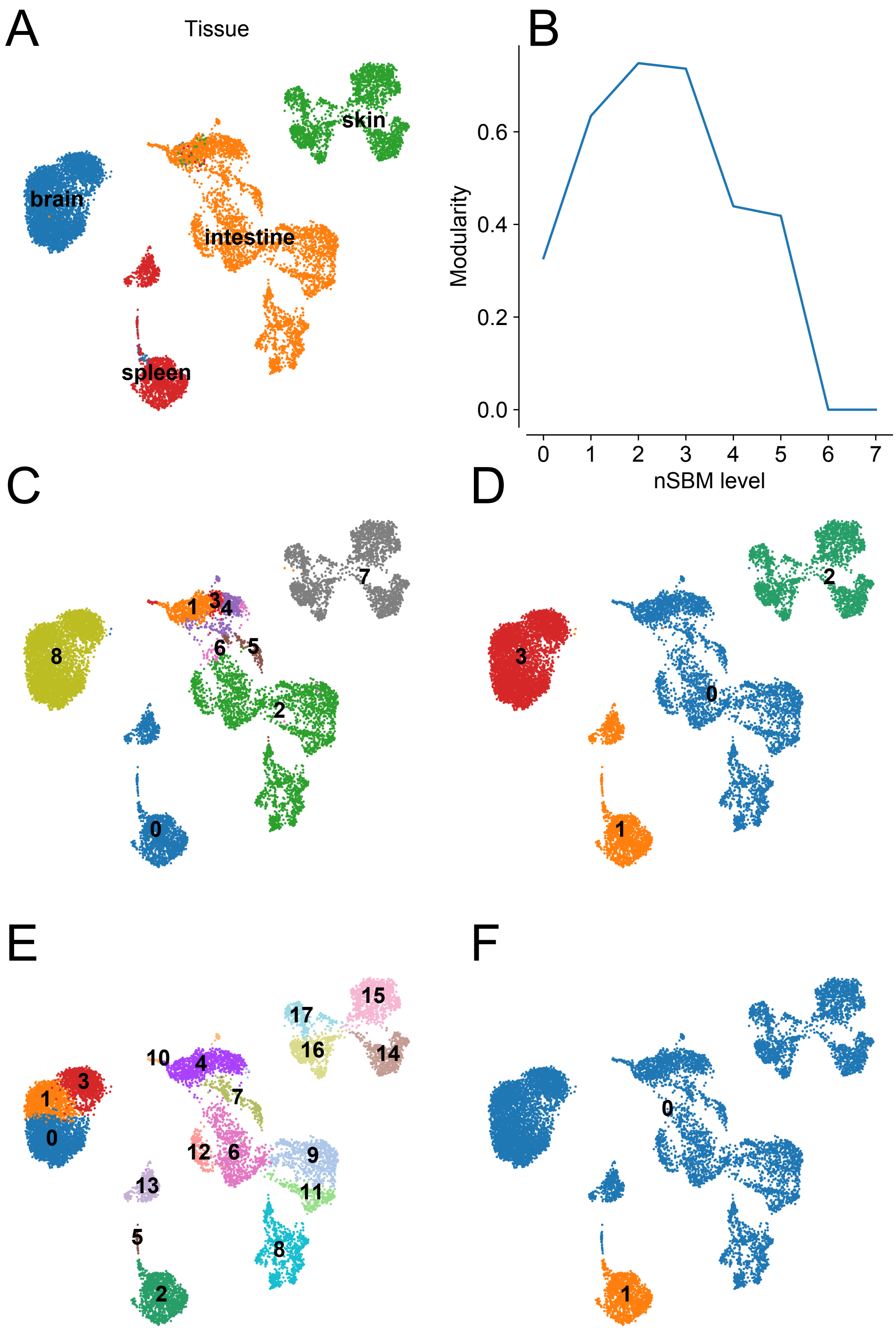

FIGURE S2: Analysis of Tabula Muris tissues. (A) UMAP embedding of single cells coloured by tissue identity. (B) Profile of the modularity at different levels of nSBM hierarchy. (C) UMAP embedding showing cells coloured by level 3 of the hierarchy proposed by the nested Stochastic Block Model. (D) UMAP embedding coloured according to SCCAF classification when partitions in (C) are used. (E) UMAP embedding showing cells coloured according to the Leiden method at resolution $\gamma = 1$. (F) UMAP embedding coloured according to SCCAF classifications when partitions in (E) are used.

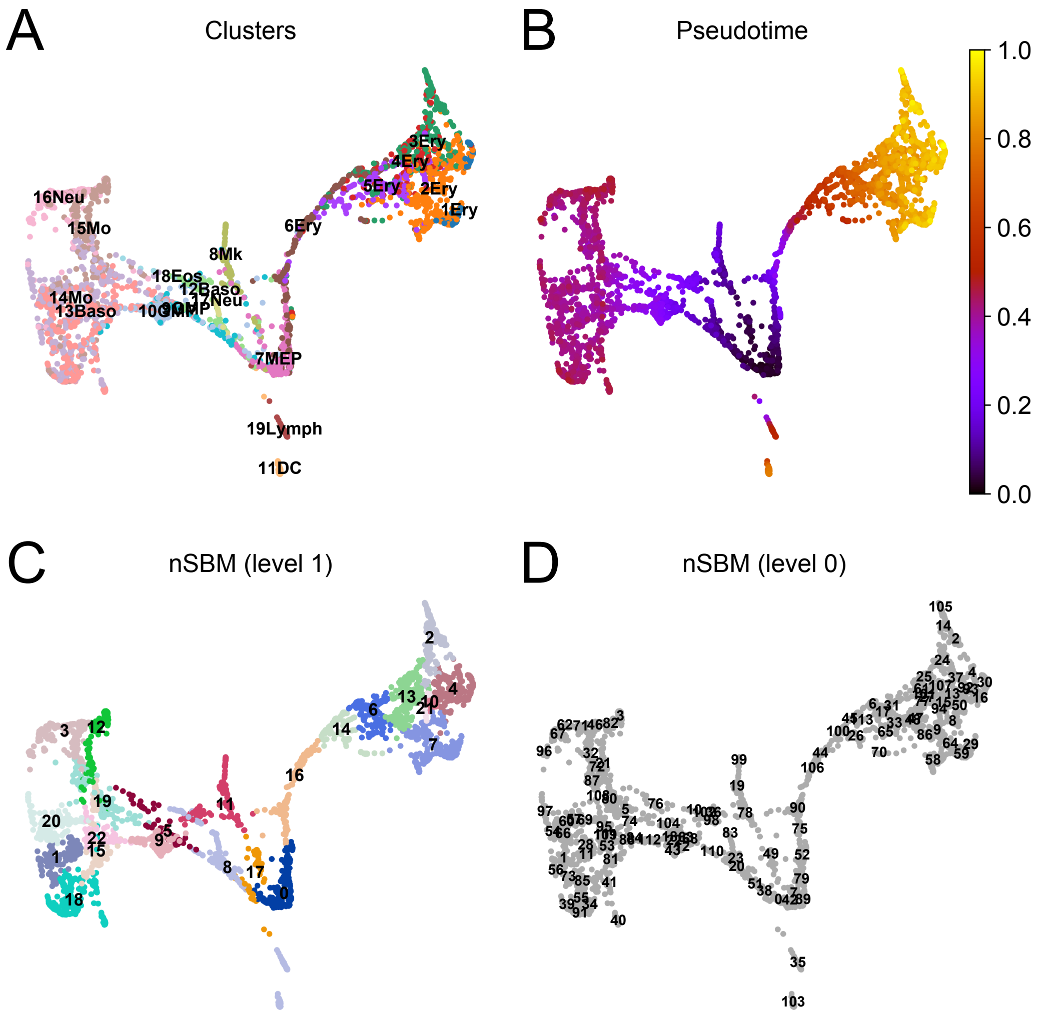

Figure S3: Low dimensional embedding of single cells from hematopoietic differentiation dataset. Cells are colored by original cell type (A), pseudotime (B), nSBM grouping at level 1 of the hierarchy (C) and level 0 (D). Since level 0 identifies 114 groups, only group labels have been represented.

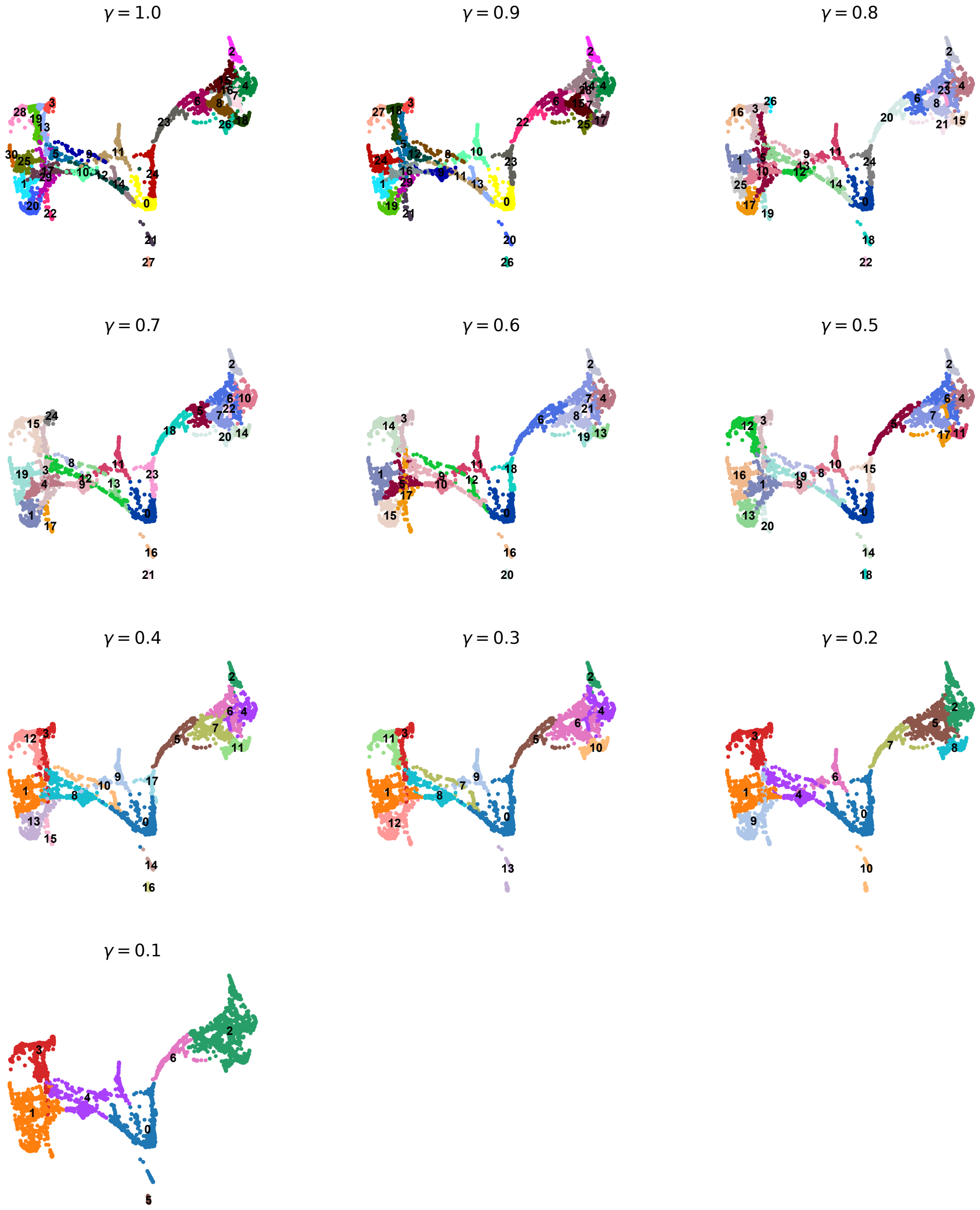

Figure S4: Low dimension embedding of single cells for hematopoietic differentiation coloured according to Leiden clustering at decreasing resolution, from 1.0 to 0.1. Lowering the distribution does not grant that cells are grouped in a hierarchical way, *e.g.* groups 6 and 8 in the Erythroid branch at resolution $\gamma = 1$ are merged or split at coarser resolutions ($\gamma = 0.6$ and $\gamma = 0.3$).

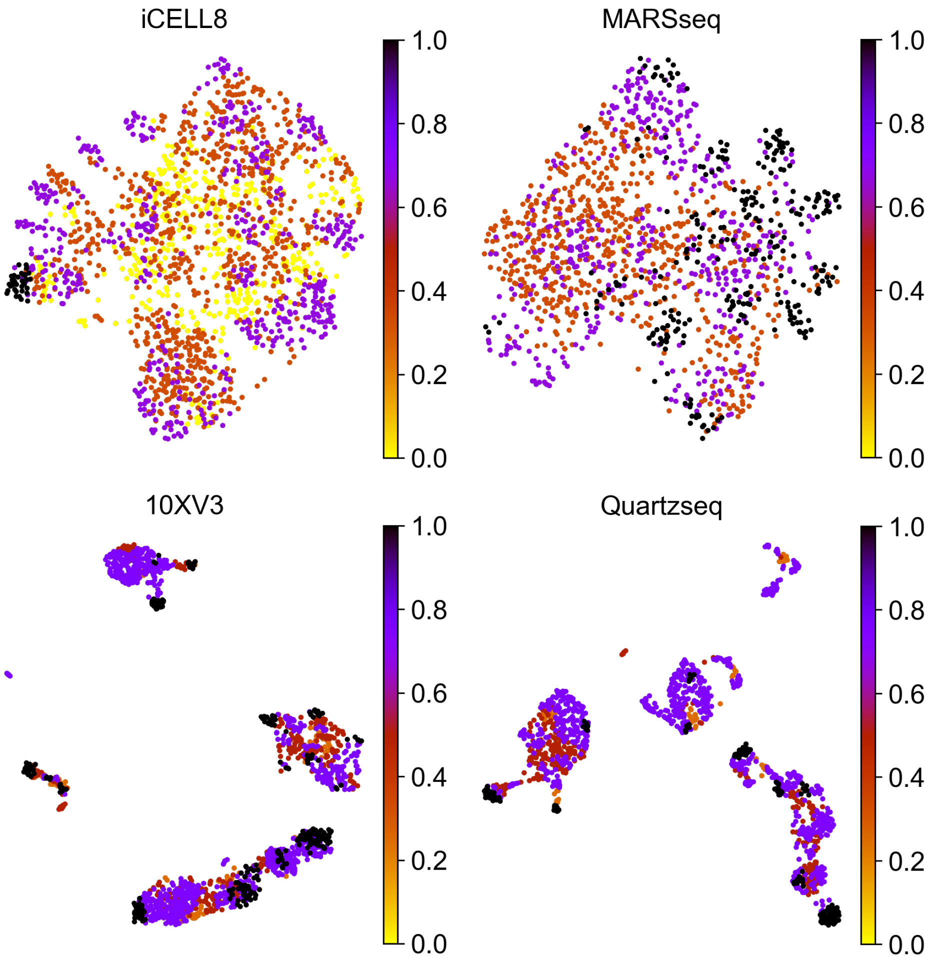

FIGURE S5: Cell Stability. UMAP embeddings of PBMC data profiled with four different technologies ranked by their quality. Cells are coloured by the Cell Stability metric.

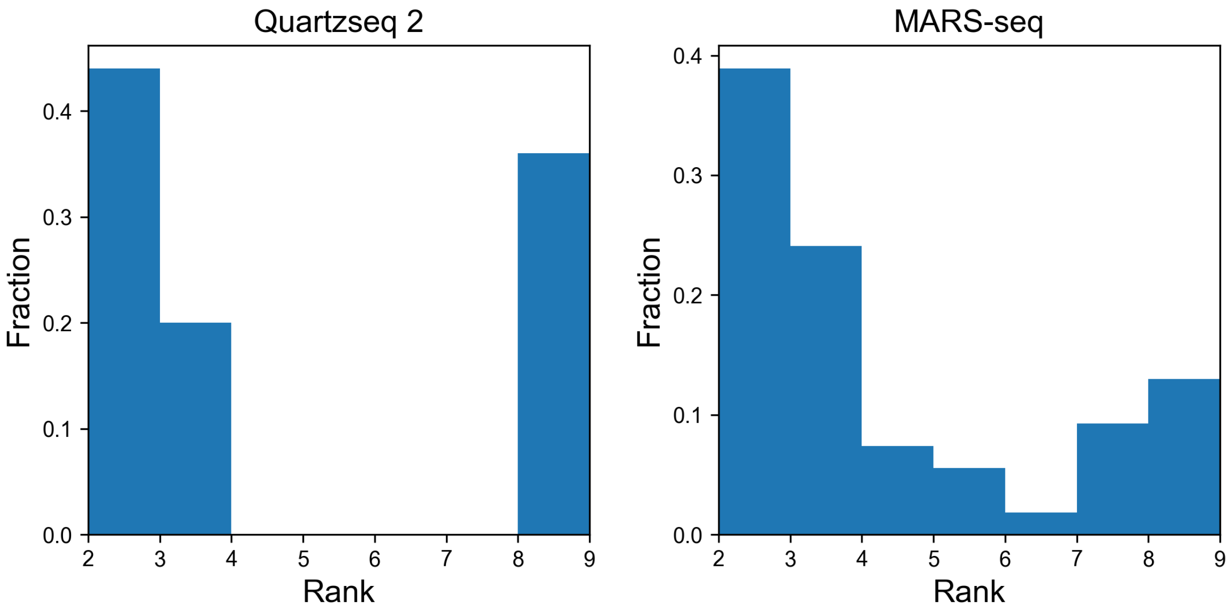

Figure S6: Distribution of second choices in label transfer. For each dataset in which we assessed accuracy of label transfer by SBM, we collect the rank of affinities of the correct class when an assignment could not be performed (*i.e.* the highest affinity was for the "Unknonwn" label)

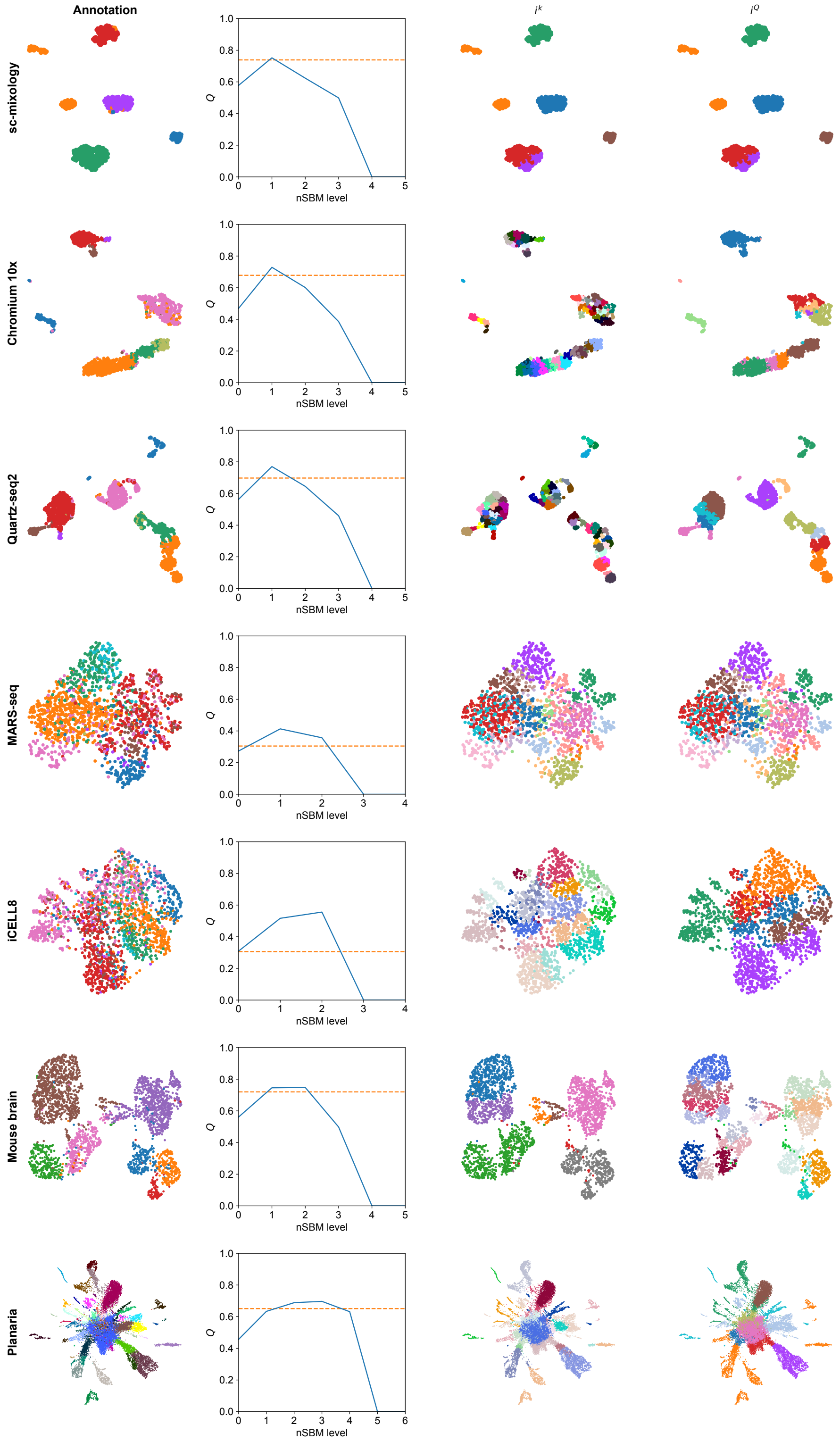

FIGURE S7: Choice of the optimal hierarchy level. Datasets analysed for choice of the most relevant hierarchy level are represented here, from top to bottom in the same order as in Table 2. For each dataset we report the UMAP embedding coloured by the annotation given in the corresponding manuscript, the profile of modularity Q at different level of the hierarchy and the UMAP embeddings coloured by the level according to i^k or i^Q. The dashed line in modularity plots represents the modularity calculated using the annotation from the corresponding manuscripts.

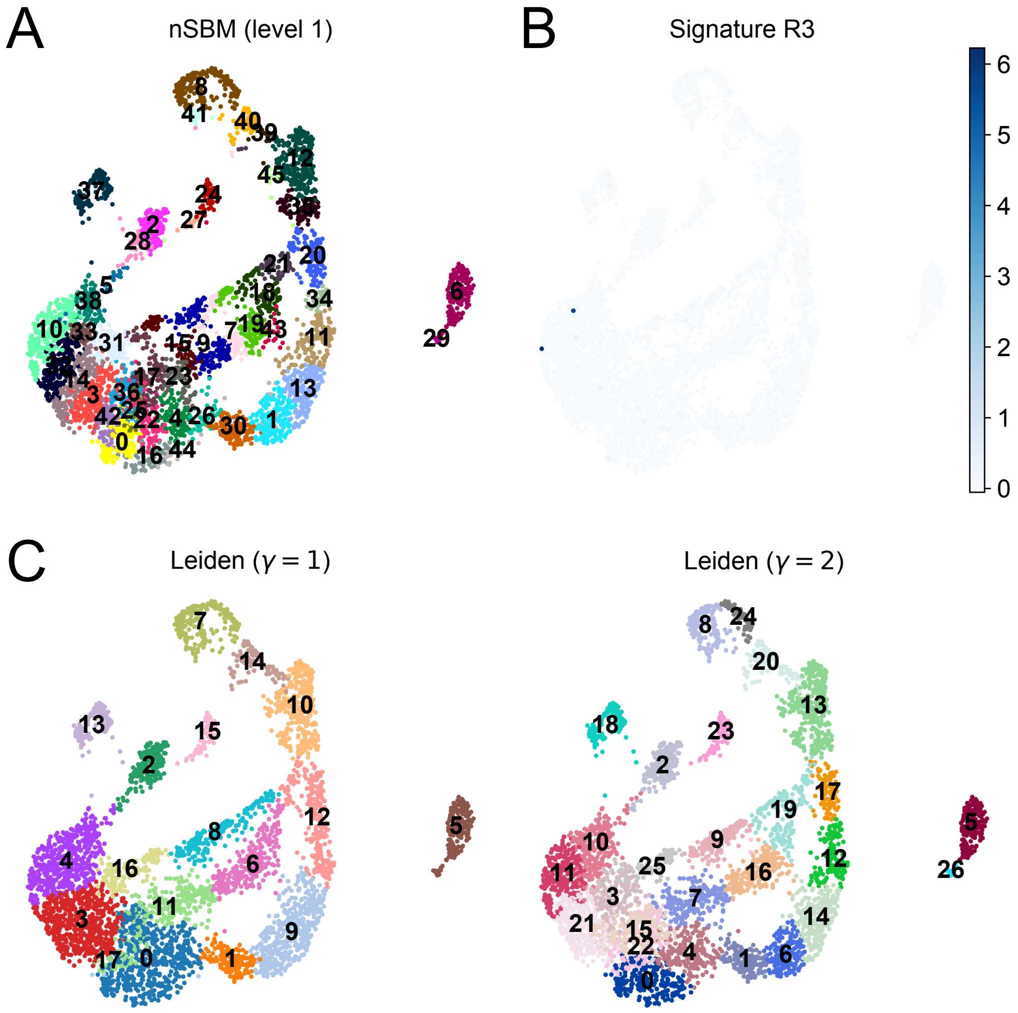

FIGURE S8: Identification of rare populations. (A) UMAP embedding of mouse crypt cells coloured by cell groups resulting at level 1 of the nSBM hierarchy. (B) Cells coloured by the signature of R3 group identified by GapClust. (C) UMAP embedding of mouse crypt cells coloured after optimisation of modularity at default parameters (left) or higher resolution (right).

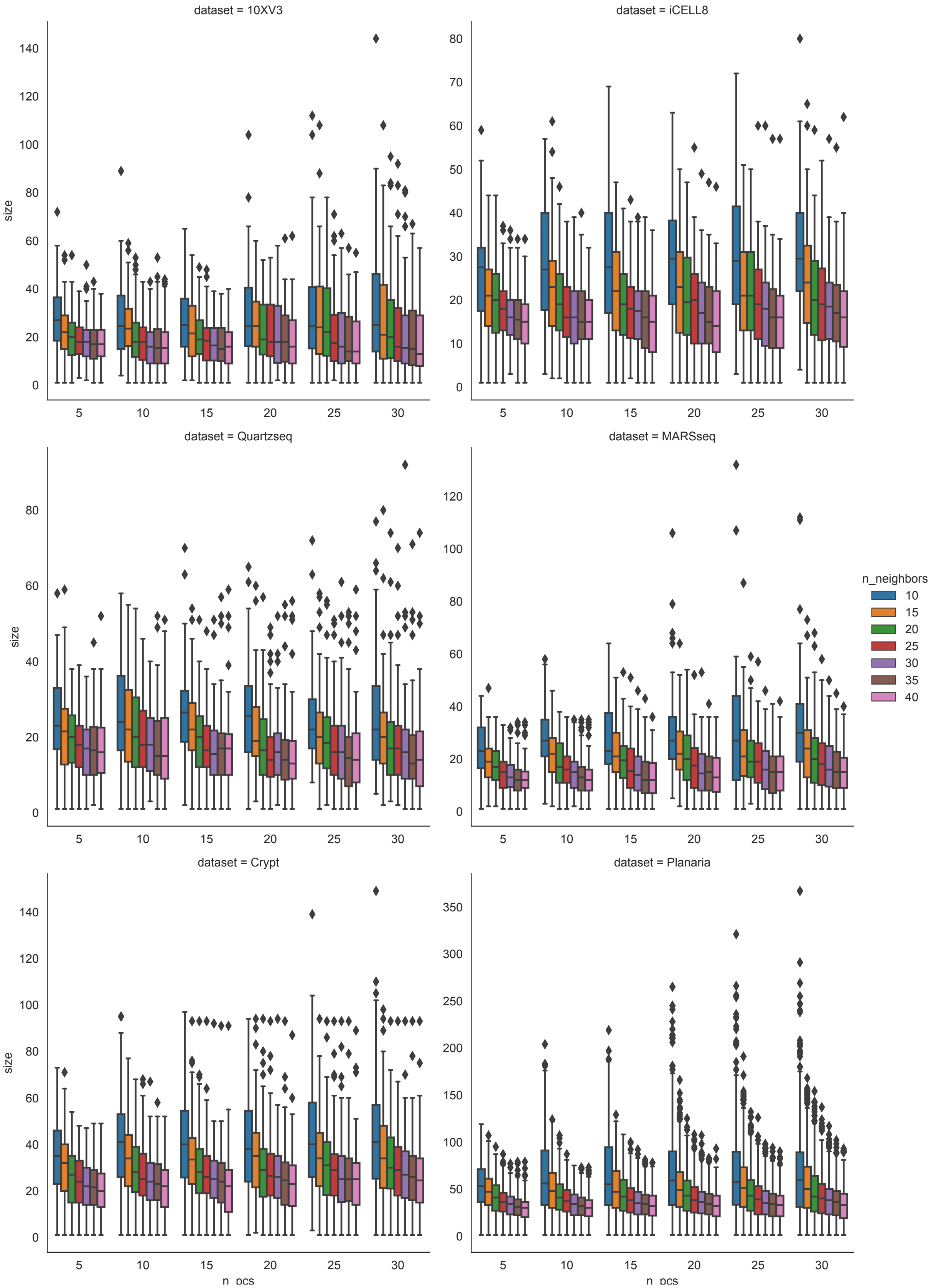

Figure S9: Evaluation of the size of communities. Boxplots show the distribution of size of communities found at level 0 of the nSBM hierarchy for various datasets used in the manuscript when different parameters are chosen to build the kNN graph. Increasing the number of neighbors results in more granular partitioning (*i.e.* smaller communities), increasing the number of principal components results in more dispersed sizes. x-axis: number of principal components; y-axis: community size; bar color: number of kNN neighbors.

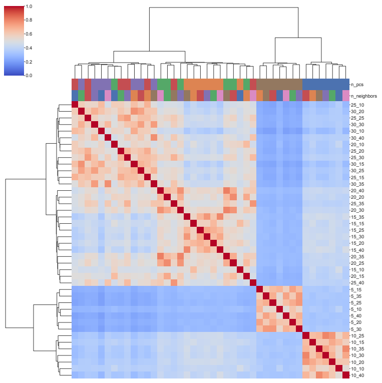

(A) Chromium 10x

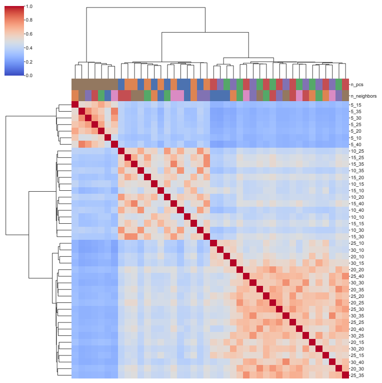

(B) iCell8

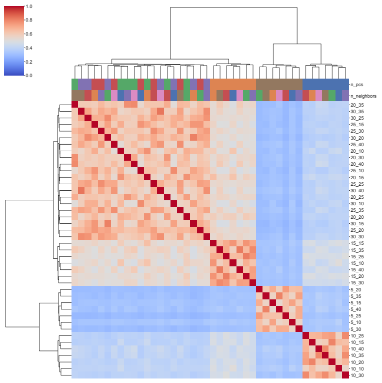

(C) Quartz-seq2

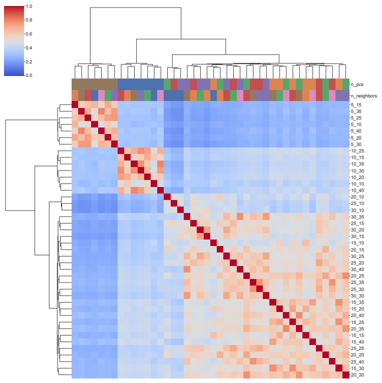

(D) MARS-seq

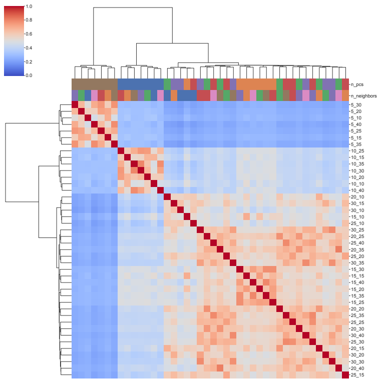

(E) Crypt cells

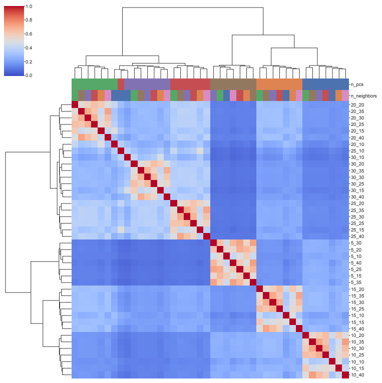

(F) Planaria

FIGURE S10: Consistency at Level 0. Each heatmap shows the Adjusted Rand Index among partitions found when different parameters are used to build th kNN graph. Row labels encode the number of PCs and the number of neighbors (P_N), reflected in colors used to annotate columns.
